# ICP-AES: A Realistic Assessment of Its Capabilities for Food Analysis

**DOI:** 10.6028/jres.093.075

**Published:** 1988-06-01

**Authors:** J. W. Jones

**Affiliations:** Center for Food Safety and Applied Nutrition, U.S. Food and Drug Administration, Washington, DC 20204

Accurate determination of trace elements in foods is a formidable challenge for even the most capable analyst. The complex and disparate matrices of foods, coupled with the often very low concentrations of nutritive or toxic elements, combine to push the capabilities of existing trace element analytical methods to their limits.

Inductively coupled plasma atomic emission spectrometry (ICP-AES) is now a widely used technique for food analysis. Na, P, Ca, Mg, Fe, Zn, Cu, Mn and Sr can be readily determined in most foods by JCP-AES. However, several other important elements such as AI, Mo, Ni, Cr, Pb, Se, As, Cd, Co, V and Hg cannot be easily determined in most foods by ICP-AES unless extensive chemical procedures are used to concentrate these elements to quantifiable levels in the analytical test solution, and to remove potentially interfering elements. Further, even with preliminary chemical separations of analytes from any given matrix, significant spectral interferences may remain, necessitating extreme care during the ICP-AES determination.

The U.S. Food and Drug Administration conducts a continuous nationwide monitoring program to estimate the dietary intake of selected food contaminants (including toxic elements) and nutritive elements, In this Total Diet Study representing the typical U.S. diets, 234 food types are prepared (e.g., peeled, cooked) as for consumption. The foods range in matrix complexity from drinking water to beef stew or chocolate cake. These 234 foods encompass the full range of “difficult” food types usually encountered in a food analysis laboratory. We have analyzed all of these foods for many of the elements above using ICP-AES to: a) estimate the approximate concentration ranges of the elements; and b) assess the capability of ICP-AES for accurate determination of some of the more difficult to determine elements such as V and Co.

During this project, we noted many problems with acid digestion procedures, separation chemistry, and the ICP-AES determinative step, all of which could adversely affect the accuracy of our determinations. Appropriate test portion sizes which are sufficient to provide enough analyte mass to quantify, but which also can be accommodated safely by the digestion procedure (nitric, perchloric, sulfuric acids) must be selected. Compatibility of the test portion size and the digestion procedure with the post-digestion chemistry (Chelex 100 ion exchange) is essential. Finally, the recognition of less common, but significant, spectral interferences for elements such as Ti is critical to obtain an accurate ICP-AES determination of V or Co.

For many of the 234 foods, it was not possible to obtain accurate ICP-AES estimates of some elements, most notably, Co and V. The concentrations of these elements were generally well below 10 ng/g (in about 75% of the foods), and in a few cases the presence of relatively high concentrations of Ti rendered useless the more sensitive ICP-AES emission lines for V and Co.

Nevertheless it was possible to estimate upper limit daily dietary intake values of these two elements. We were able to quantify Mo, Ni and Cd in most of the samples. Based on these analyses, we have estimated the U.S. daily dietary intakes. These estimates are expressed in ranges to include the various age/sex groups in the U.S. population. They are, in micrograms per day: Mo, 50-126; Ni, 69-162; Co, 3-12; V, 6-18; and Cd, 8-15.

These preliminary estimates clearly illustrate the very low intakes of elements such as Co, V, Cd, Mo, and Ni and the corresponding need for extreme care by the analyst in reporting low-level findings in foods. The potential exists for serious errors in ICP-AES determinations of these elements. This is illustrated in figures 1–3 for Co and V.

Figures 1 and 2 show two of the more commonly used ICP-AES Co emission lines. In figure 1, the Co line at 238.9 nm is seriously overlapped by an Fe line. Depending on the quality of the spectrometer and the experience of the analyst, the interference could quite conceivably go unnoticed, resulting in a gross overestimation of the Co levels in foods. The analyst who is able to recognize this serious Fe interference might elect to avoid it by selecting another Co emission line at 228.6 nm, a line with approximately equal sensitivity. For most foods, this line is adequate for obtaining at least upper-level limits for Co. However, in the presence of high Ti levels such as those found in some processed foods containing Ti additives, the line is rendered useless by a direct Ti overlap interference (fig. 2). Titanium is also a potentially major interference for V at 292.4 nm (fig. 3), Such interferences may easily go undetected even by an experienced ICP-AES analyst, since Ti is rarely determined in foods and the analyst has no obvious reason to suspect that the emission intensity observed at the Co or V lines is not, in fact, Co or V.

These examples serve to illustrate the care needed when ICP-AES is used to determine trace elements in foods. Highly experienced analysts in trace element chemistry and ICP-AES spectrometry are essential when ICP-AES is used to estimate these types of dietary intakes.

ICP-AES can be used successfully to estimate the levels of selected nutritive and toxic elements in foods. However, despite the low determination limits possible with ICP-AES, a great deal of effort is required to obtain consistently reliable results. These efforts include the use of relatively large test portions to obtain enough analyte element for quantitation, separation chemistry to concentrate the elements to quantifiable levels, contamination control, ICP-AES instrumentation capable of characterizing interferences, and highly experienced ICP-AES analysts who recognize interferences and other inherent limitations of the technique.

## Figures and Tables

**Figure 1:**
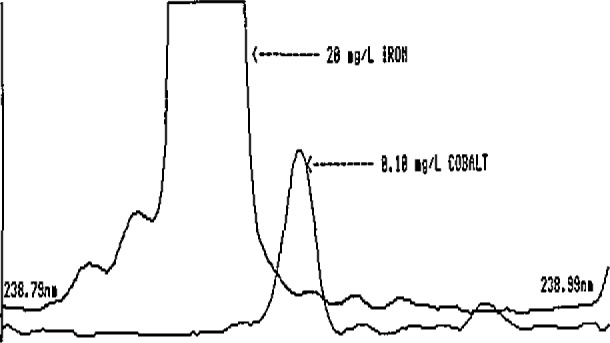
Spectral line overlap interference of iron on the 238.9-nm line of cobalt.

**Figure 2:**
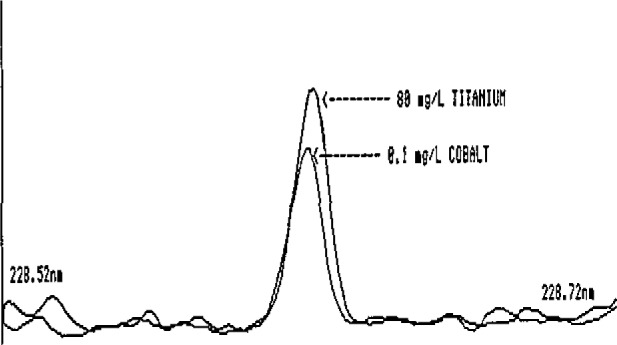
Spectral line overlap interference of titanium on the 228.6-nm line of cobalt.

**Figure 3:**
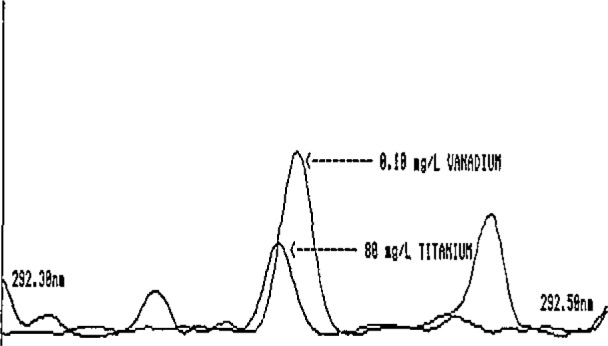
Spectral line overlap interference of titanium on the 292.4-nm line of vanadium.

